# Frugal-Oriented Information and Communication Technology for Development Framework Toward Low-Cost Digital Maternal Health in Low- and Middle-Income Countries: Quantitative Descriptive Study

**DOI:** 10.2196/77330

**Published:** 2026-04-27

**Authors:** Ronald Danny Nyatuka, Md Shafiqur Rahman Jabin, Lisa Dionne-Morris

**Affiliations:** 1 School of Computing and Engineering Sciences (SCES), Strathmore University, Nairobi, Kenya; 2 Department of Medicine and Optometry, Linnaeus University, Pedalstråket 11, Kalmar, 392 31, Sweden, 44 7915 673 61; 3 School of Mechanical Engineering, Faculty of Engineering and Physical Sciences, University of Leeds, Leeds, United Kingdom

**Keywords:** digital health innovation, maternal health promotion, underserved populations, sub-Saharan Africa, innovative methods and strategies

## Abstract

**Background:**

The Sustainable Development Goals (SDGs) aim to eradicate poverty and inequality while ensuring that all individuals enjoy good health. Among these, target 3.1 seeks to reduce the global maternal mortality ratio to less than 70 per 100,000 live births. However, progress toward this target has been limited, particularly in low- and middle-income countries (LMICs), where health care delivery remains constrained by limited resources. While digital innovations have increasingly been adopted to improve health care access and service delivery, a significant proportion of populations in LMICs continues to experience inadequate access to essential maternal health services. This gap underscores the need for affordable, sustainable, and contextually appropriate strategies that are cost-effective in improving maternal health outcomes in underserved communities.

**Objective:**

This study leverages the principles of frugal innovation and information and communication technologies for development (ICT4D) to propose a frugal-oriented ICT4D framework to deliver low-cost digital maternal health solutions in LMIC settings. The framework seeks to optimize the use of available resources, foster equitable access to maternal health care, and contribute toward achieving SDG 3, particularly target 3.1.

**Methods:**

The study was conducted in both rural and urban-poor settings in Kenya using a 2-phased quantitative approach. In phase 1, eight theoretical themes relevant to maternal health uptake were explored. These themes were represented on color-coded sorting cards, which participants ranked according to perceived importance. Phase 2 involved administering structured survey questionnaires to collect empirical data. The study included a total of 32 participants, whose insights provided a foundation for analyzing the significance of contextual factors influencing maternal health service utilization.

**Results:**

The weighted scores for 3 of the 8 predetermined theoretical themes—such as resources, information services, and social support programs—emerged as the most influential factors shaping maternal health promotion (N=32). Resources ranked highest (n=6, 18.81%), followed by information services (n=6, 17.99%), while social support programs accounted for 9.64% (n=3) of the overall influence. These findings highlight critical enablers and barriers within the maternal health care landscape and provide a nuanced understanding of contextual dynamics that affect the uptake of maternal health services. The results informed the design of a frugal-oriented ICT4D framework that prioritizes low-cost digital interventions tailored to resource-limited settings.

**Conclusions:**

Despite increasing recognition of digital innovations as tools for health care transformation in LMICs, adoption of existing capital-intensive solutions remains low due to financial and infrastructural constraints. This study emphasizes the importance of adopting frugal innovation and ICT4D principles in designing low-cost, scalable digital health interventions to improve access to maternal health care. Implementing such approaches can address resource limitations, enhance maternal health outcomes, and support progress toward SDG 3, particularly target 3.1. The proposed framework provides a foundation for future research and practical interventions aimed at sustainable, equitable maternal health service delivery in LMIC contexts.

## Introduction

### The Context of Maternal Mortality and Sustainable Development Goals

The overall aim of the Sustainable Development Goals (SDGs) agenda is to transform our world; hence, it is a call to action to end poverty and inequality, protect the planet, and above all, ensure that all people enjoy good health [1]. Unfortunately, the world faces myriad threats, including conflict, climate change, and the aftermath of the COVID-19 pandemic, which are devastatingly impacting the progress made so far in reducing poverty toward achieving the SDGs [2]. This has led to an increase in the number of people and households living in extreme poverty, thus causing the most significant rise in between-country inequality. There is a wide disparity between high-income countries and low- and middle-income countries (LMICs), leading to inequitable global development. LMICs have continued to lag in fiscal and overall capacity relative to their high-income country counterparts, due to more significant vulnerabilities among LMIC communities [3].

Notably, a significant portion of the global population, including women and children, lacks access to essential health care services, leading to poor health outcomes. Over 287,000 women died in 2020 due to pregnancy and childbirth-related complications [4], which translates to about 800 women dying every day during the same period [2]. In addition, millions of children have been killed globally from preventable causes, including poverty, disease, and hunger [5]. On a global scale, an estimated 774 million children live in multidimensional poverty, while at the same time, 100 million people are pushed into poverty annually due to high health care costs. As a result, childhood vaccinations have declined substantially over the past 3 decades, whereas deaths from tuberculosis and malaria have increased since the COVID-19 pandemic [2].

Unfavorable socioeconomic conditions, including income levels, employment status, gender, education, and ethnicity, influence the situation. These factors push individuals to lower socioeconomic status, thus exposing them to a higher risk of poor health [6]. Therefore, it is against this background that target 3.1, whose objective is ‘‘to reduce the global maternal mortality ratio to less than 70 per 100,000 live births,” was established. The SDG agenda commits to reducing maternal mortality to achieve universal health coverage (UHC) by 2030 [2]. There is a need to address setbacks through greater innovation and investment to build resilient health systems to withstand future health threats, including pandemics like COVID-19. This could be achieved by leveraging relevant innovative approaches and information and communication technologies (ICTs) to enable social and economic development toward achieving the SDGs.

### ICTs for Development (ICT4D) and Frugal Innovation in Health Care

ICT4D refers to using ICT in various domains, including socioeconomic development, international development, and human rights, to bridge the digital divide and improve the lives of the underserved in LMICs [7]. ICT4D in health care, therefore, entails the use of ICTs to collect, store, retrieve, and exchange health care information to facilitate efficient, effective, and patient-centered care [8-11] to enhance socioeconomic development. However, the application of ICT4D in Kenya’s health sector is not exceptional in the face of earlier barriers, particularly inadequate health infrastructure, leading to high-cost health care ICT innovations [12]. There is a lack of funding to provide sufficient health ITs, including computer hardware and software, skilled personnel [10,13], and an appropriate policy framework to facilitate the optimum utilization of ICTs in health care delivery [14-16]. To address these challenges adequately, the principles of ICT4D (digital development) and frugal innovation (FI) should be leveraged. ICT4D principles include designing with the user; understanding the ecosystem; designing for scalability; building for sustainability; data-driven solutions; using open standards, open data, open source, and open innovation; reusing and improving ICT tools; and addressing privacy and security concerns while strengthening collaboration among key stakeholders [7,17].

Frugality is one way to produce effective and affordable health care innovations using fewer resources to deliver services to marginalized populations [18]. FI is a strategy about ‘‘how to do more with less’’ to overcome resource constraints and develop novel and locally relevant solutions to address local problems [19,20]. It refers to products, services, and systems that can be redesigned and re-engineered for underserved users to facilitate access to basic services and infrastructure [16]. The concept of FI entails the application of the principles of simplicity, affordability, and sustainability in the design and implementation of digital technologies, which has potential to strengthen health systems for better health care delivery and management in LMICs, such as sub-Saharan Africa (SSA) [21-23]. The benefits of ICT4D can therefore be leveraged through combining business, IT systems, and social innovation through redesigning or re-engineering business processes and products (hardware and software) with new features in digital applications using minimal resources. These resources can be easily recycled for increased value addition, with a focus on cofunctionalities (simplicity), cost-effectiveness (affordability), and maintainability (sustainability) to mitigate resource constraints in providing digital services to the public [24-26] to make them more readily accessible. A perfect example of FI in the context of ICT4D in Africa is the high mobile penetration, which helps bridge the digital divide by providing consumers with access to services. Notably, the simplicity and affordability of mobile phones have led to increased adoption rates of mobile financial services, such as M-PESA, in Kenya and within SSA countries [27,28].

ICT4D solutions can help address local health concerns and promote long-term values for communities. Therefore, ICT4D encompasses the use of ICT across the fields of socioeconomic development, international development, and human rights [7]. It is a form of digital development that uses and applies digital tools and solutions to address social, economic, and environmental challenges [29,30]. The significance and potential of ICT4D lie in its focus on aiding underserved and impoverished populations by empowering them with access to information, services, opportunities, and rights. Increased ICT access and use have significant potential to accelerate the attainment of the SDGs, as highlighted in recent analyses of the ICT4D value chain [31,32].

### Study Aims and Research Questions

To establish sustainable health care systems in LMIC settings, it is necessary to embrace frugal ICT4D innovations in the health sector. The concept of FI should be adopted to strip down, simplify, and create efficient digital health innovations to increase access to health services for marginalized and underserved groups [14,33-35]. This calls for resource-constrained approaches to innovation, such as frugal digital innovation (FDI) in LMIC settings. The paper aims to leverage the principles of FI and ICT4D to propose a novel frugal-oriented ICT4D innovation framework to guide the development of low-cost digital innovations for underserved communities in Kenya. Specifically, the study seeks to explore contextual factors influencing maternal health in underserved contexts, such as Africa, to inform the proposed solution. This will help to increase access to health care, with a focus on maternal health in SSA toward achieving SDG 3.1 [24,34]. The research questions to be answered by the study include the following:

- What contextual factors influence the utilization of digital maternal health services among underserved communities in LMIC settings?
- How could low-cost digital maternal health interventions be developed to promote maternal health among underserved communities in LMICs?

## Methods

The study, conducted between March and July 2023 in 2 contextual sites, rural and urban-poor settlements, in Kenya, is significant as it focuses on the uptake of maternal health in LMICs, a crucial aspect of public health. The main target was maternal health stakeholders in the public health sector, including pregnant women and health care practitioners.

### Study Design

#### Overview of the study

A quantitative approach was used because of its immense benefits in improving the validity of research. This included its ability for objectivity in measurement and data collection, use of standardized instruments to reduce researcher bias, identification of trends and patterns, generalizability of findings, empirical evidence to support decisions, as well as the replication and comparison of findings [36-38]. Despite the limitations of quantitative research approaches in capturing the uncertainty of complex relationships and interactions among research phenomena, the study used theoretical themes to supplement the surveys.

#### Sampling Procedure

A total of 32 participants were recruited for the study, comprising 2 main groups: 15 pregnant women and 17 professional stakeholders. Participants were drawn from the designated study sites, namely rural Kitui County and the urban-poor Kibra informal settlements in Nairobi.

Pregnant women were recruited based on their willingness to participate, age eligibility, and residency within the selected study locations. Professional participants, including community health providers (CHPs), were nominated from various stakeholder groups operating within the study sites. A snowball sampling technique was employed to identify additional eligible participants [39,40].

The inclusion criteria for professional stakeholders included age eligibility, relevant educational qualifications (minimum of postsecondary education), employment in a maternal health–related role (eg, CHPs, health policy experts, or ICT specialists), and work experience. Participants were also required to be affiliated with a facility or organization serving the study communities during the research period. These criteria were clearly communicated to initial key informants and applied consistently throughout the snowball sampling process. Detailed inclusion and exclusion criteria for each stakeholder group are provided in Table 1.

**Table 1.** Detailed inclusion criteria by stakeholder category^a^.

Stakeholder category	Inclusion criteria
Pregnant women (service users)	Age: above 18 years Residency: residing in either a rural setup within Kitui County or a poor-urban setup within Nairobi’s Kibra Slums during the study period Consent: willingness to participate in the study
Health professionals (nurses, clinical officers, medical officers, and midwives)	Age: above 18 years Educational qualifications: have a minimum of a postsecondary qualification (diploma or higher) in nursing, midwifery, medicine, or a related health discipline Role: employed as a health care worker providing maternal health services Experience: have a minimum of 3 years of work experience in the current or a related role Affiliation: should be working in a health facility or organization serving the study communities
CHPs^b^	Age: above 18 years Education: have at least a secondary school level education Role: be accredited CHPs actively serving in the catchment communities for ≥1 year Recruitment: be identified and nominated via snowball sampling techniques Experience: have a minimum of 1 year of active engagement in maternal health–related community support
Health policy and ICT^c^ specialists (technical)	Age: above 18 years Educational qualifications: have a minimum of a postsecondary qualification (diploma or higher) in a relevant field (either in public health or health management and IT/ICT or computer science), respectively Role (health policymakers): be a specialist in maternal health policy, occupy a supervisory/managerial role (eg, county health management team member or facility in charge), and in a related field Role (ICT specialists): be supporting health-related ICT systems/programs Experience: have a minimum of 3 years in a professional role in a relevant field

a The table outlines the eligibility and inclusion criteria used to purposively select study participants across stakeholder categories involved in maternal health service delivery, community support, and health policy and ICT systems.

b CHPs: community health providers.

c ICT: information and communication technology.

#### Execution of the Study

The study was conducted in two phases: (1) an exploration of 8 predetermined theoretical themes as potential contextual factors for maternal health in LMICs and (2) quantitative survey research.

#### Exploration of Theoretical Themes

**Figure 1.**
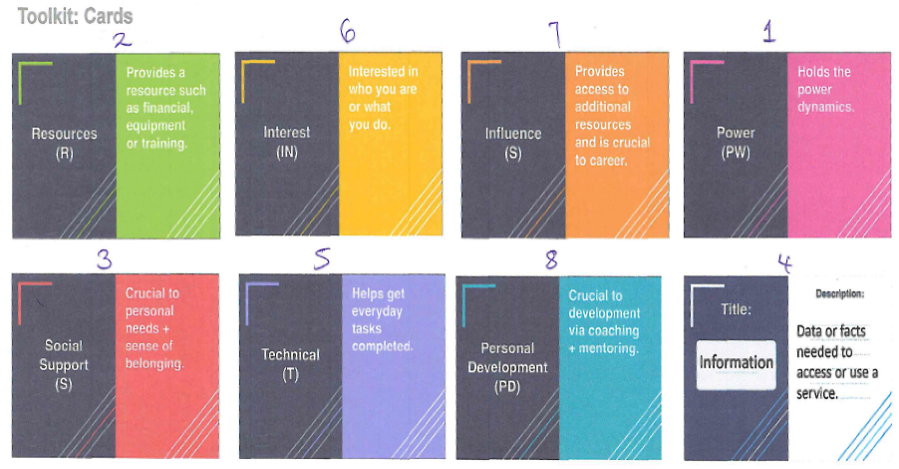
Illustration of participants ranking sorting cards representing contextual factors influencing maternal health in low- and middle-income countries (LMICs), arranged according to individual preferences.

Stage 1 of the study was based on theoretical themes identified as potential individual and contextual factors influencing the uptake and utilization of maternal health services in an African context, including education, poverty, population density, media exposure, autonomy, empowerment, and access to health care services [41,42]. Against this background, 8 themes were derived from the literature and explored to guide the study. They include information, resources, social support, technical competency, personal development, power, interest, and influence. Using a toolkit of sorting cards to represent the various themes in different colors as shown in Figure 1, the researchers selected 13 out of the 32 participants randomly to rank the factors (1-8) according to the individual order of preference. This would assist researchers in identifying potential priority areas in enhancing the utilization of maternal health services to guide the study.

#### Quantitative Survey Study

The design and development of survey questionnaires were tailored to the various participant groups in the study, based on the most significant factors influencing maternal health utilization in the study context. This was achieved through the sorting cards ranking exercise. Questionnaires were developed with closed-ended questions and administered to all participant groups, that is, pregnant women, health professionals, health policymakers, CHPs, and ICT experts.

### Data Analysis and Interpretation

#### Sorting Cards

The sorting of the card data was analyzed based on individual stakeholder participants’ ranked order of the 8 contextual factors perceived to influence the utilization of digital maternal health services, as per the card-sorting grid presented in Table 2.

**Table 2.** Ranked card-sorting results showing participants’ prioritization of contextual factors influencing digital maternal health utilization from highest to lowest.

Participant	1	2	3	4	5	6	7	8
NCW1	Information	Resources	Power	Technical competency	Interest	Influence	Personal development	Social support
NCW2	Information	Resources	Interest	Influence	Power	Technical competency	Social support	Personal development
NHP 1	Resources	Power	Technical competency	Interest	Information	Influence	Personal development	Social support
NHP 2	Resources	Information	Personal development	Interest	Influence	Technical competency	Social support	Power
KHP 1	Information	Resources	Social support	Personal development	Interest	Technical competency	Influence	Power
KHP 2	Resources	Information	Interest	Personal development	Information	Social support	Influence	Power
KHP 3	Resources	Technical competency	Interest	Information	Social support	Personal development	Influence	Power
KCW1	Power	Resources	Social support	Information	Technical competency	Interest	Influence	Personal development
KCW2	Power	Resources	Social support	Personal development	Information	Influence	Technical competency	Interest
PMSP1	Information	Technical competency	Resources	Power	Interest	Influence	Personal development	Social support
PMSP2	Information	Technical competency	Resources	Interest	Influence	Personal development	Social support	Power
PM1	Technical competency	Social support	Influence	Personal development	Interest	Power	Resources	Information
HP1	Information	Resources	Personal development	Technical competency	Social support	Interest	Influence	Power

#### Ranking and Selection of Important Factors

Participants’ rankings placed resources and information as the top 2 contextual factors, followed by technical competency, interest, power, social support, and personal development (see Table 3). These results informed the selection of resources, information services, and social support programs as the focal dimensions for the survey.

**Table 3.** Weighted importance of factors influencing the utilization of maternal health services^a^.

Factor	Weighted score, %
Resources	18.81
Information	17.99
Technical competency	12.85
Interest	11.99
Power	10.06
Personal development	9.85
Social Support	9.64
Influence	8.78

a Percentage values were calculated by dividing each factor’s weighted score by the total score (467) and expressing the result as a proportion of 100%.

The various contextual factors were ranked and prioritized in terms of their respective scores as represented in Table 3. The subsequent quantitative survey, however, focused on 3 out of the 8 contextual factors, that is, resources, information services, and social support programs, respectively. Resources and information were selected because they received the highest overall rankings (positions 1 and 2) in the card-sorting exercise and therefore represented priority structural determinants of digital maternal health utilization in this setting. Social support programs, although ranked seventh, were included because they were found to be consistent with empirical evidence that low social support during pregnancy is associated with poorer mental health and adverse outcomes. For example, the Bedaso et al’s systematic review and meta-analysis report that low social support is significantly associated with antenatal depression and anxiety among pregnant women. Participants’ responses also emphasized the importance of social protection mechanisms, including cash transfers, health insurance, and other support services, in enabling pregnant women to access and sustain maternal health service utilization. Together, these 3 dimensions offered a conceptually coherent and contextually grounded set of priorities to guide the design of the survey instrument and subsequent analysis.

### Quantitative Survey

#### Survey Instrument Development and Administration

Survey questions were based on the 3 selected factors to guide the study: resources, information services, and social support programs. The data were analyzed and presented using descriptive statistics, including percentages and tabulations. Data analysis was based on the 3 factors, as illustrated in subsequent sections.

#### Resources

.Among the 15 pregnant women surveyed (N=15), the utilization of maternal and child health services was distributed across multiple service areas. Antenatal care, child immunization, general medical care, and nutrition services were the most frequently accessed, each reported by 2 (13.3%) respondents, as shown in Table 4. In contrast, postnatal care, baby delivery, medical laboratory, pharmacy, health education, family planning, and child welfare services were each accessed by 1 (6.7%) respondent.

**Table 4.** Distribution of maternal health services^a^.

Maternal health service(s)	Values, n (%)
ANC^b^	2 (13.3)
PNC^c^	1 (6.7)
Baby delivery	1 (6.7)
Child immunization	2 (13.3)
General medical care	2 (13.3)
Medical laboratory	1 (6.7)
Pharmacy	1 (6.7)
Nutrition	2 (13.3)
Health education	1 (6.7)
Family planning	1 (6.7)
Child welfare	1 (6.7)

a Percentages were calculated based on 15 respondents and redistributed proportionally to total 100%.

b ANC: antenatal care.

c PNC: postnatal care.

Overall, the findings indicate a relatively even distribution of service utilization, with no single service overwhelmingly dominant. This pattern suggests that pregnant women attending public community health facilities engage with a broad range of maternal and child health services, reflecting the multifaceted nature of care-seeking during pregnancy.

#### Information Services

The study examined 2 key components within the maternal information dimension: “information needs” and “information sources.”

#### Information Needs

With respect to information needs (N=15), 4 major elements were identified: diet and nutrition, pregnancy-related complications, causes of miscarriage, and physical fitness and exercise. Diet and nutrition and pregnancy-related complications emerged as the leading areas of concern, each accounting for 28% (n=4) of responses. These were followed by causes of miscarriage and physical fitness and exercise (n=3 each), while other categories were minimally reported at 2% (n=1) (see Table 5).

**Table 5.** Distribution of maternal health information needs for pregnant women^a^.

Type of information need	Percentage score, %
Diet and nutrition	28
Pregnancy-related complications	28
Physical fitness and exercise	24
Causes of miscarriage	18
Others	2

a Percentage scores represent the proportion of respondents reporting each category as a key maternal health information need.

These findings indicate that information related to diet and nutrition, as well as pregnancy-related complications, represents the most critical knowledge areas for supporting preventive maternal health care among pregnant women. The prominence of these needs underscores the importance of implementing more targeted and effective interventions, particularly digital information services, to facilitate faster, secure, and timely dissemination of maternal health information among relevant stakeholders.

The majority of respondents (11/15, 80%) reported that they received relevant information, while 20% (3/15) indicated that they only occasionally received the required information. However, when assessing the adequacy of the information received, 27% (4/15) of women reported that their information needs were only partially met, and 13% (2/15) indicated that their needs were not met at all. These findings suggest that although access to information is relatively high, gaps remain in ensuring that maternal health information is sufficiently comprehensive and responsive to women’s specific needs.

#### Information Sources

A total of 32 participants reported that information services supporting maternal health promotion programs were derived predominantly from human sources (n=21, 67%), including health professionals, CHPs, and family members. In contrast, nonhuman sources, such as books, magazines, and the internet (n=2, 6%), accounted for a smaller proportion of information channels. Notably, health IT tools were not reported as a source of maternal health information (n=0, 0%) as shown in Table 6.

**Table 6.** Distribution of sources of maternal health information for expectant mothers in the community^a^.

Source of information	Percentage score, %
Health professionals	25
CHPs^b^	19
Family and friends	23
Mass media	17
Books, magazines, and pamphlets	10
Internet	6
Health IT tools	0

a Percentages represent the proportion of respondents reporting each source as a channel for receiving maternal health information.

b CHPs: community health providers.

According to the study findings, 94% of maternal health information was primarily accessed through traditional sources, including paper-based books and magazines, verbal communication among patients, health professionals, CHPs, and family members, as well as mass media (Table 5). In contrast, internet-based sources accounted for only 6% of responses, while health IT tools were not utilized at all (0%). These findings indicate minimal adoption of digital health technologies or the nonexistence of the relevant infrastructure within the study setting.

With regard to the use of ICT for accessing, sharing, and communicating maternal health information, respondents (N=14) reported varying levels of computer literacy: 67% (n=9) indicated being computer literate, 13% (n=2) reported partial computer literacy, and 20% (n=3) were computer illiterate. The most commonly used digital devices and platforms included mobile phones (n=4, 31.9%), SMS text messaging intervention services (n=3, 23.4%), and television and radio (n=3, 19.4%). Email and social media accounted for 10.6% (n=1) of usage, whereas desktop computers and laptops were least utilized (n=0, 2.1%). These findings suggest that although basic digital literacy is relatively high, the use of advanced digital health tools remains limited, with mobile-based technologies serving as the primary medium for digital communication.

#### Social Protection and Support Services

The findings illustrate the distribution of social protection services available to pregnant women and children within the study context as shown in Table 7. Food relief and health insurance constituted the most prominent forms of support, followed by cash assistance, security services, and children’s assistance. Subsidized housing represented the least provided social protection service among those assessed, as shown in the table.

**Table 7.** Distribution of primary social protection services offered to pregnant women^a^.

Social protection service	Percentage of responses, %
Cash assistance	19
Food relief	22
Subsidized housing	5
Health insurance	21
Security	17
Children assistance	16

a Field survey data on primary social protection services offered to pregnant women and children in the study area.

The findings indicate that social protection efforts mainly prioritize immediate needs, particularly food security and health insurance, reflecting a focus on short-term welfare and protection against health-related financial risks. However, lower provision of subsidized housing and moderate levels of cash, security, and child assistance suggest gaps in addressing structural determinants of maternal and child well-being. Strengthening integrated, long-term social protection strategies is necessary to enhance economic stability, safe living conditions, and sustainable health outcomes.

### Ethical Considerations

The study received ethical approval (reference: MEEC21-029) from the University of Leeds Research Ethics Review Committee in the United Kingdom on November 14, 2022, prior to its commencement. All relevant ethical considerations concerning key stakeholders, including the researcher, research participants, and the sponsoring organization, were carefully addressed, and the study strictly adhered to these guidelines to ensure compliance and integrity throughout the research process. In addition, informed consent was obtained from all participants before participation, and their confidentiality and privacy were safeguarded through the coding of responses to conceal their identities. Participants were also compensated for their time and any inconvenience or expenses incurred during the study period, including transport and airtime costs.

## Results

### Key Contextual Factors Influencing Maternal Health Service Utilization

This study highlights the complex contextual factors influencing pregnant women’s access to and utilization of digital maternal health services in public community health facilities within underserved LMIC settings. Using weighted scores derived from sorting cards across predetermined theoretical themes, the analysis identified resources (18.81%), information services (17.99%), and social support programs (6.64%) as the most salient dimensions. These findings provide a blueprint for designing frugal ICT4D solutions aimed at promoting equitable access to maternal health care. Participants ranked resources and information as the most significant constraints on service utilization, ranking them first and second, respectively (see Table 3). Although social support was ranked seventh, it was deliberately retained for 2 reasons. First, its theoretical importance is well established, as prior studies indicate that social support strongly influences maternal and perinatal health outcomes as well as care-seeking behavior in LMICs [43,44]. Second, its contextual relevance is supported by stakeholder feedback, which highlights the critical role of social protection programs, such as cash transfers, food relief, and health insurance, in mitigating poverty-related barriers to health care access in Kenya.

We therefore selected resources, information, and social support as the focal dimensions for the survey because they combine high empirical salience with strong theoretical and contextual relevance for maternal health promotion in underserved Kenyan settings. By integrating these structural and social dimensions, the study provides a comprehensive understanding of the multifaceted challenges shaping maternal health service utilization in underserved LMIC contexts.

### Resources

The study observed that the dimension of “resources” was critical and a strong influencer of the utilization of maternal digital health services in the study setting. Despite the crucial role of digital technologies in promoting maternal health services to women during the pregnancy period, it greatly depended on the availability of necessary resources. These included the physical infrastructure itself, as well as equipment, devices, and services needed for health facilities and households to operate efficiently. Notably, the needed internet infrastructure and access to and affordability of computing equipment and devices, such as smartphones, laptops, and desktop computers, played a key role toward the utilization of digital maternal health services by stakeholders. Particularly, digital access to such services, including health insurance, recommended nutritional guidelines, and general health education programs, was equally important as far as maternal health promotion was concerned. For instance, out of 14 women, only 10.6% (n=2) of them had access to email and social media, whereas 2.1% (n=0) owned a desktop/laptop, and 31.9% (n=5) had access to a smartphone. Furthermore, the majority of women were not covered by the National Health Insurance Fund to access maternal health services in government health facilities, hence a lack of UHC. Therefore, in underserved LMIC settings, new strategies should be developed to address resource scarcity and promote equitable access to maternal digital technologies, thereby enhancing health care delivery.

### Information Services

**Figure 2.**
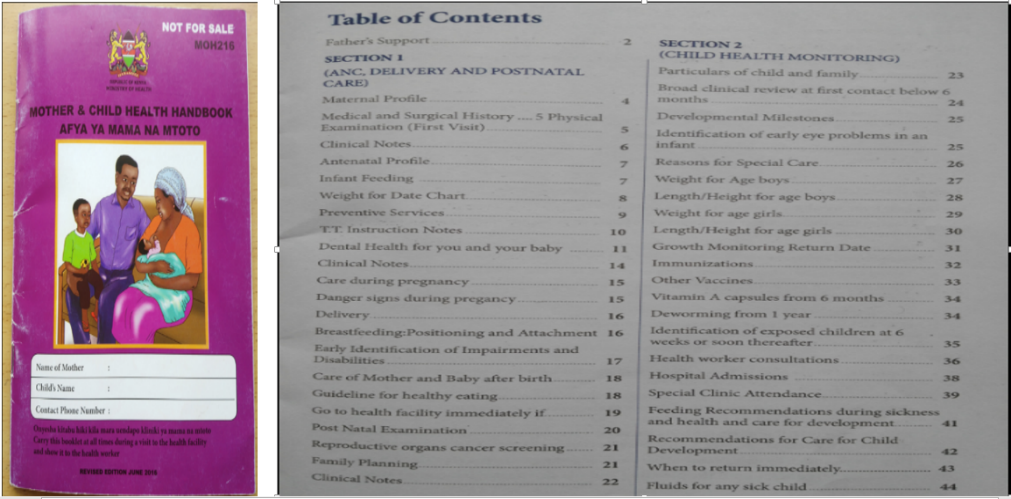
Antenatal care (ANC) booklet with maternal profile contents.

The study observed 2 significant categories of information services provided by the government to promote and utilize maternal health services/programs. These include maternal health information through the Ministry of Health and information on existing social protection services through the Directorate of Social Protection. These categories of information were considered vital for promoting and utilizing maternal health programs among pregnant women, underscoring the importance of the findings. The study noted that the nature of the information services offered by the 2 government departments was mainly paper-based/manual, with a smaller percentage in digital form. Paper-based information services were prone to faster deterioration and destruction, thus losing vital patient health data/information. Women participants expressed frustration regarding the mother and child booklet (shown in Figure 2), the only government-approved standard document containing the individual maternal profile.

**Figure 3.**
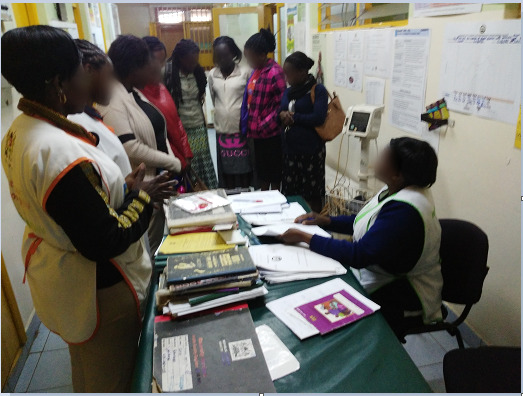
Pregnant women attending an antenatal care (ANC) health education session at a local community health facility.

The booklet was used to manage and share maternal health information, including recording clinical notes and serving as a reference for clients and health professionals during antenatal visits and at the point of care. However, the booklet was prone to loss or destruction by natural causes, such as fire outbreaks [14]. Generally, paper-based information services and sources were the order of the day as shown in Figure 3. The major limitation of manual records is that they are prone to faster deterioration by agents of destruction, leading to the loss of vital patient information and fragmentation of health information records and systems.

### Social Support Services

To alleviate the vulnerability of pregnant women/mothers and children from poor households, the government initiated social protection programs/services to supplement maternal health promotion services, including the Nutrition Improvements through Cash and Health Education initiative program. The program provided cash assistance and transfers to enable pregnant women and nursing mothers to access basic needs, such as food and health care.

## Discussion

### Digital Solutions to Address Resource Limitations

Our study findings confirm the existence of resource-limitation challenges arising from inadequate digital health care infrastructure, which is insufficient to meet the needs of women and children [14]. Long queues at health facilities indicate an acute shortage of both health infrastructure and personnel to address the demand for maternal health services. Therefore, there is a critical need to provide affordable, cost-effective, timely, secure, and efficient digital information services to promote maternal health.

Affordable digital solutions have the potential to significantly enhance access to maternal health promotion services, including health education and telemedicine. Such interventions can facilitate lower-cost consultations and support better personal care decisions, ultimately improving health outcomes for pregnant women and children in marginalized communities within LMICs. These improvements can be realized through the 3 key dimensions of maternal health promotion: resources, information, and social support services.

### Resources

Among the reasons for partial or incomplete access to services are long patient queues in public facilities, long geographical distances between the area of residence and the health facility, poor or lack of transport infrastructure, and the unaffordability of health services for women [14,45]. The majority of the women were merely homemakers, and therefore, their low economic status meant that most of them could only afford health services to a limited extent. The situation calls for finding ways to address resource constraints and enable equitable access and utilization of maternal health services toward enhancing UHC [3].

### Information Services

The findings confirm a lack of integration and collaboration among different stakeholders and thus information silos, making sharing and communication of health information difficult [46,47]. This situation underscores the need for digital maternal health information services, rather than traditional forms, to better address fragmentation. The dimensions of information needs and information sources played a critical role in determining the nature of information services provided, whether manual or digital.

### Social Support Services

According to the findings, such programs, including relief food, health insurance, and cash transfer services, were most needed, suggesting high levels of poverty in many households. This situation increases vulnerability to poverty, hence exposing pregnant women and children to the risk of poor health care during pregnancy and postnatal care. Understanding the importance of these social support services will help you empathize with pregnant women’s challenges.

### Proposed Research Framework

**Figure 4.**
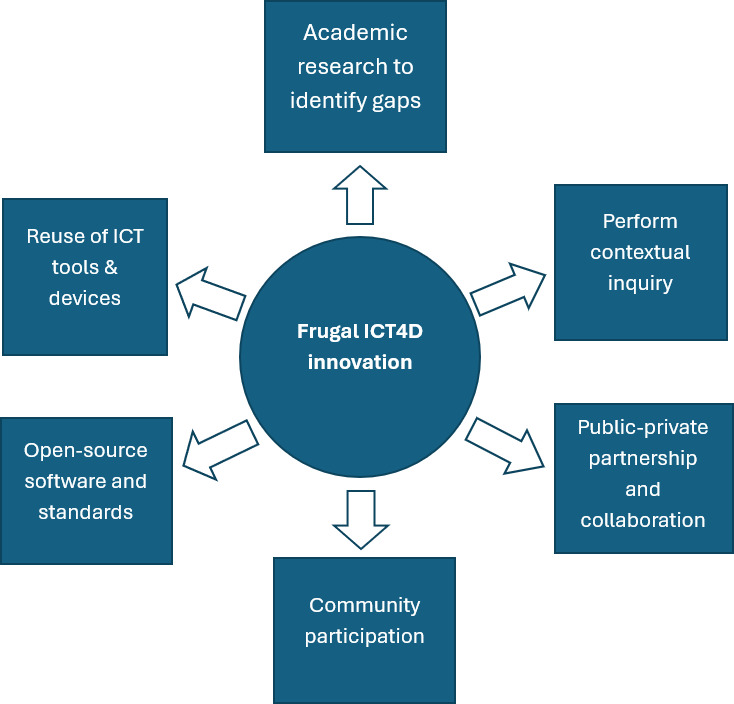
Frugal-oriented information and communication technologies (ICTs) for development (ICT4D) innovation framework.

Based on the study findings, the proposed solution (frugal-oriented ICT4D innovation framework) comprises six dimensions, namely (1) academic research and development (R&D), (2) contextual inquiry, (3) public-private partnership (PPP) and collaboration*,* (4) community participation, (5) open-source software and standards, and (6) reuse of ICT tools and devices as shown in Figure 4. The conceptual model is aligned with the principles of FI and ICT4D to create sustainable, low-cost digital innovations that promote the delivery of maternal health services to underserved populations in LMIC settings.

### Academic R&D

R&D is a critical requirement for innovation toward economic development [48]. Academic research should be leveraged to identify gaps in ICT4D for health, tap students’ skills and staff expertise to deliver sustainable, low-cost digital health interventions in frugal settings, and promote maternal health. IT/information systems research projects, community ICT4D initiatives, and industrial attachments for graduate and undergraduate students should be leveraged under the supervision of senior academic staff to deliver affordable digital innovations. This will help lower the costs of developing or acquiring digital health solutions for the purpose of affordability [35] rather than hiring full-time personnel. Frugal ICT4D innovation emphasizes the need to build intersections between technical, social, and institutional innovations, particularly on how their material properties enable the FDI processes [49].

### Contextual Inquiry

ICT4D research studies should be contextualized to deliver context-specific innovations that maximize impact in the context of use [50]. This enables the use of what is locally available and takes into consideration critical contextual factors, including people’s culture, socioeconomic situation, technological infrastructure, etc, which facilitate considerable savings [49], while at the same time creating meaningful ICT4D innovations that are responsive and fit into existing work practices and social context.

### PPP and Stakeholder Collaboration

A PPP and health stakeholder collaboration with clear business obligations between public and private partners should be set out under constitutional and contractual terms to raise the necessary resources to develop products and services [21,51]. It involves working collaboratively among different stakeholders and partners (individuals, groups, and organizations) who may have specific interests in the project [52]. Particularly, internal stakeholders (health professionals and providers/hospitals) and external stakeholders (government, patients, and community workers) should be engaged to alleviate predominant resource constraints by contributing, in the form of sharing, their inputs in terms of knowledge, skills, and resources [53]. This will involve industry-academia and community collaborations focusing on underserved population groups and communities in LMICs to achieve sustainable community development through ICT4D innovations [54]. Collaboration enables open innovation for the public sector, which requires governments to listen more closely to citizens and other end users of digital innovations who are the beneficiaries of new public services. This helps overcome barriers to open innovation, such as immature methods for participation, inadequate understanding of how to formulate appropriate strategies for developing public sector innovations, institutional barriers to innovation and collaboration, weak innovation culture, poor funding, and a technology-deterministic approach to innovation [49].

### Community Participation

The involvement of community members is critical for planning, decision-making, implementation, and monitoring of health projects that directly affect their health and well-being [8]. The approach enables context-specific initiatives by empowering local people and ensuring that development efforts are tailored to the community’s local needs and priorities, including positive health outcomes [41]. Through community participation, members build relationships with public institutions and individuals, giving their community access to valuable external resources, such as funding and social support, thereby strengthening social capital.

### Open-Source Software and Standards

Given the underserved nature of communities in LMIC settings, with predominant resource constraints, free, that is, open-source, software tools should be optimized, such as Ushahidi crowdsourcing, which allows users to collect, analyze, and visualize information at a lower cost [47,55]. Open-source tools and standards are attractive to FDI because they are free to download and use, can be optimized, and significantly reduce software development and acquisition costs to address resource limitations, thereby positively impacting the community. Moreover, the open-source software easily conforms to open standards and is thus universal, unlike proprietary software [34,49].

### Reuse of ICT Tools and Devices

According to Peyal et al [33], FDI implies a scenario in which innovators harness the power of readily available digital technologies to develop new products and services that use fewer resources to deliver greater value. This entails using ICT tools and devices with frugal characteristics to make them inexpensive [49], which involves reusing what is available to minimize wastage to ensure resource efficiency. ICT adoption significantly influences FI, such that greater levels of frugality in ICT4D innovations increase user satisfaction [56]. To reduce the cost of development or acquisition, there is a need to reuse, combine, and recombine computing resources (software, hardware, and networks) to create new products and services. This fosters affordability and accessibility of digital health services within contextual constraints, delivering economic and social values [49,57].

### Study Limitations and Future Research

The selection of the key informants was mainly performed purposefully, based on the role(s) of each participant in the research context, including health care policymakers, pregnant women/mothers, health professionals, CHPs, and ICT experts. Other essential criteria might not have been considered. Furthermore, the study was mainly conducted in government health facilities and thus excluded the private sector, where some of the participants received health care services. This may have otherwise provided good responses and insights into the study, enabling comparison and improving the validity of the findings. In addition, it is essential to note that the study findings were based on stakeholders’ responses on maternal health from the perspectives of resources, information, and social protection services in health facilities in Nairobi and Kitui counties. To validate the findings outside the study contexts, future studies should cover a broader geographical scope by extending the study to more counties and including both public and private facilities. The relevance of these study findings outside the original context is not guaranteed; hence, there is a need to verify them empirically across various underserved settings in LMICs.

### Conclusion

Underserved communities from LMIC settings, such as SSA, live in abject poverty, a scenario that consequently denies the majority of individuals and households a chance to access basic health care, including pregnant women and children. This is attributable to the inability to afford health care services, which compromises health and well-being and places marginalized communities at higher risk of poor health outcomes. Despite efforts to create digital innovations to enable health promotion toward achieving SDG 3, little progress has been made, particularly in maternal health. An estimated 800 women die globally every day from pregnancy and childbirth-related complications, some of which cases are preventable. There is low adoption of existing expensive digital health innovations, and thus, they lack cost-effectiveness in an LMIC context. Therefore, these calls for adopting cost-effective approaches, such as FI and ICT4D, to redesign and create sustainable, low-cost digital innovations to mitigate resource constraints and deliver greater value. The proposed research framework can serve as a blueprint to guide the design and implementation of sustainable low-cost ICT4D innovations to increase access to and promote health, including maternal health, for the underserved in LMICs toward achieving SDG 3.

## Supplementary material

10.2196/77330 Multimedia Appendix 1 Data presenting the diet sheet, suggested stakeholders to be involved in proper maternal health care, and stakeholder mapping exercise.

## Abbreviations

CHPs: community health providers
FDI: frugal digital innovation
FI: frugal innovation
ICT: information and communication technology
ICT4D: information and communication technology for development
LMICs: low- and middle-income countries
PPP: public-private partnership
R&D: research and development
SDGs: Sustainable Development Goals
SSA: sub-Saharan Africa
UHC: universal health coverage
